# Evaluation of TGFβ, XPO4, elF5A2 and ANGPTL4 as biomarkers in HCC

**DOI:** 10.3892/etm.2012.750

**Published:** 2012-10-16

**Authors:** HAO ZHANG, SHI WEI, SONG NING, YUNGLIU JIE, YUKANG RU, YUCHUN GU

**Affiliations:** 1 Department of Surgery, Huashan Hospital, Fudan University, Shanghai 200040;; 2 Institute of Molecular Medicine, Peking University, Beijing 100871, P.R. China

**Keywords:** hepatocellular carcinoma, XPO4, TGFβ1, ANGPTL4, elF5A2, biomarker

## Abstract

Hepatocellular carcinoma (HCC) is the most common type of liver cancer, and the fourth leading cause of cancer mortality worldwide. It is often diagnosed at an advanced stage, and hence typically has a poor prognosis. A number of distinct molecules have been recently identified as playing a role in the control of cancer progression. However, patients with HCC have a highly variable clinical course, indicating that HCC comprises several biologically distinctive subgroups reflecting a molecular heterogeneity of the tumors. To evaluate potential biomarkers in HCC, we employed multiple methods in this study, including qPCR, immunostaining methods and tissue microarrays (TMAs), as well as histological and pathological analysis, to assess TGFβ, XPO4, elF5A2 and ANGPTL4 in cancerous and paracancerous liver tissues from 280 patients suffering from liver cancer. Our results found that all four indicators were located in the cytoplasm and distributed in cancerous and paracancerous liver tissues. Generally, there were higher levels of these indicators in paracancerous, compared with cancerous, liver tissues. These four indicators were correlated and modulated among each other. In connection with patient clinical and revisit information, statistical analysis determined that TGFβ1 in paracancerous liver tissue was positively correlated with tumor size. Higher production of TGFβ1 in paracancerous liver tissue was always associated with bigger liver tumors. XPO4 in cancerous liver tissue and TGFβ1 in paracancerous liver tissue were positively correlated with tumor differentiation. TGFβ1, ANGPTL4 and elF5A2 were also positively correlated with the T classification of tumors. Additionally, higher levels of XPO4 in cancerous liver tissue suggested that the patient would have a better prognosis and survival rate. However, higher production of XPO4 in paracancerous liver tissue suggested a worse prognosis. All the results above provide new insights into better understanding biological indicators, such as XPO4, TGFβ1, ANGPTL4 and elF5A2, in the prediction and evaluation of liver cancer, as well as signaling pathways in the control of liver cancer. XPO4 and TGFβ1 may serve as useful markers to evaluate the size and prognosis of liver cancer.

## Introduction

Hepatocellular carcinoma (HCC) is one of the most common malignant tumors worldwide with an extremely high incidence and poor survival rate (1,2). The management of patients at risk for developing HCC remains an intricate process. Despite the large number of studies devoted to the immunohistochemistry of HCC, at the present time, the definitive positive and negative markers for HCC are lacking.

Several key molecules in signaling pathways involved in cancer development have emerged. TGFβ plays an important role in the regulation of cell growth and differentiation, angiogenesis, extracellular matrix formation, immunosuppression and cancer development (3). It is well known that signaling by the TGFβ family is most prominent at the interface between normal tissue development and cancer. The TGFβ signaling pathway is activated upon ligands binding to type I and II trans-membrane receptors. The Smad4 protein is the downstream mediator of TGFβ. Phosphorylation of Smad, by activation of TGFβ receptors, results in activation of a TGFβ-targeted gene. ELF associates with SMAD3, presenting it to the cytoplasmic domain of the TGFβ receptor complex; this has been found to play a pivotal role in TGFβ signaling (4). Dysfunction of TGFβ pathway members, including TGFβR2, SMAD3, SMAD4 and ELF, may lead to progenitor/stem cell deregulation and possibly cancer formation. Previous studies have suggested that Smad3 and its phosphorylation relatives may be used as biomarkers to identify patients with a high risk of recurrence (5,6). Smad3 is exported via XPO4. XPO4 is therefore in control of Smad3 signaling as well as protein synthesis (7,8). A recent study indicated that XPO4 may be involved in the progression of human HCC and may serve as a potential target for gene therapy in its treatment (9). We consequently selected XPO4 as an indicator in the current study.

HCC recurrence commonly occurs with an extremely poor prognosis. Vascular invasion in HCC is one of the key factors that results in cancer recurrence. To invade, HCC cells must penetrate the vessel wall, which consists of endothelial cells and extracellular matrix components, including fibronectin and fibrinogen. TGFβ specifically phosphorylates integrinβ1 via Smad-2 and Smad-3, causing a conformational change of the extracellular component with an inside-out mechanism (10). Additionally, a previous study in breast cancer revealed that the induction of ANGPTL4 by TGFβ via Smad disrupts vascular endothelial cell-cell junctions, increases the permeability of lung capillaries and facilitates the trans-endothelial passage of tumor cells (7,11–14).

Therefore, we employed multiple methods to assess TGFβ, XPO4, elF5A2 and ANGPTL4 in cancerous and paracancerous liver tissue samples obtained from 280 patients suffering from liver cancer. We aimed to determine whether these four indicators may become biomarkers to evaluate HCC and provide an improved prognosis.

## Patients and methods

### 

#### Patients and samples

Samples were obtained under informed consent from 280 patients with HCC who underwent surgery to remove liver cancer between 2005 and 2011 in our hospital (Fudan University, Shanghai, China). All cases met the criteria set by the University of California, San Francisco (UCSF) (15). The follow-up of cases was at a mean of 42 months (range, 3–84 months). In patients with multi-nodular tumors, tumor samples were obtained from the largest tumor. Ethical approval was obtained from the Hua Shan Hospital Research Ethics Committee.

#### Tissue microarray (TMA) arrangement

TMA blocks were constructed as described previously (16). Briefly, all HCC tissues were reviewed by two histopathologists. Representative tumor areas free from necrotic and hemorrhagic materials were premarked in the paraffin blocks. Two cores, 1.5 or 2.0 mm in diameter, were taken from each representative tumor tissue, and from liver tissue adjacent to the tumor, and transferred from the recipient paraffin block at defined array positions. Six TMA blocks were constructed. Consecutive sections of 4-*μ*m thickness were taken on 3-aminopropyltriethoxysilane-coated slides (Shanghai Outdo Biotech Co., Ltd., Shanghai, China).

#### qPCR

RNA isolation and qRT-PCR was used. Total RNA was extracted from frozen tumor specimens and matched liver tissue adjacent to the tumor from 16 HCC patients using TRIzol reagent (Invitrogen Life Technologies, Carlsbad, CA, USA) according to the manufacturer’s instructions. XPO4, TGFβ1, ANGPTL4 and elF5A2 mRNA expression in tissues from these patients was measured by qRT-PCR using an IQ5 instrument (Bio-Rad, Hercules, CA, USA). qRT-PCR was performed using a SYBR PrimeScript RT-PCR kit (Takara Bio, Inc., Shiga, Japan) according to the manufacturer’s instructions. GAPDH was used as an internal control. The primers were as follows: XPO4 (Genbank NM_022459.4) forward primer 5′-TTGTTCTTGGTGTTTTGTGTTTCC-3′ and reverse primer 5′-CATTCCTTTCCCACTCCTCTT TAG-3′; TGFβ1 (Genbank NM_000660.3) forward primer 5′-GCAACAATTCCTGGCGATACCT-3′ and reverse primer 5′-CAGTGTGTTATCCCTGCTGTCACA-3′; ANGPTL4 (Genbank NM_001039667.1) forward primer 5′-GACCAA GGGGCATGGAGCTT-3′ and reverse primer 5′-CAGGGG ACC TACACACA ACAG CA-3′; el F5A 2 (G enba n k NM_020390.5) forward primer 5′-TTGTTCTCAGGG CTATTTGTGCTAA-3′ and reverse primer 5′-GGATGCTAC TGTTTCCATTTTTTTC-3′; and GAPDH (Genbank NM_002046.3) forward primer 5′-TCCCTCAACATTGTC AGCAA-3′ and reverse primer 5′-AGCTCCACAACGGAT ACATT-3′. Relative mRNA levels were calculated based on the Ct values and corrected for GAPDH expression, according to the equation: 2^−ΔCt^ [ΔCt = Ct (target gene) - Ct (GAPDH)]. All experiments were performed in triplicate.

#### Immunostaining

The primary antibodies used for immunohistochemistry were XPO4 (polyclonal rabbit, diluted 1:100; PAB0297, Abnova, Taipei, Taiwan), TGFβ1 (monoclonal mouse, diluted 1:3,000; MAB2505, Abnova), ANGPTL4 (monoclonal mouse, diluted 1:200; L191591e, Enzo, NY, USA) and elF5A2 (polyclonal rabbit, diluted 1:30; E9781, Sigma, St. Louis, MO, USA). Immunohistochemistry was carried out using a two-step method as described previously (16). Following heat-induced antigen retrieval, tissues were incubated with primary antibodies for 30 min at room temperature. Following a 30-min incubation with a matched secondary antibody, sections were developed in 3,3′-diaminobenzidine solution (Sigma) under microscopic observation and counterstained with hematoxylin (Sigma). Negative control slides in which the primary antibodies had been omitted were included in all assays.

#### Statistical analysis

Analysis was performed with SPSS 17.0 for Windows (SPSS Inc., Chicago, IL, USA); the values are expressed as the mean ± standard error. A two-tailed P-value of <0.05 was considered to indicate a statistically significant difference. The differential expression of proteins between carcinoma tissue and adjacent tissue was determined by a t-test. In statistics, correlation refers to any of a broad class of statistical relationships involving dependence. The most common method is the Pearson correlation coefficient (CC), which is sensitive only to a linear relationship between two variables. Two variables are more correlative if the CC value is closer to 1. Relationships between clinicopathological and molecular parameters were statistically analyzed using Pearson or Spearman’s rank correlation coefficients. P<0.05 indicates that the two groups are correlative. Kaplan-Meier analysis was used to determine survival. The log-rank test was then used to compare patient survival between subgroups.

## Results

### Distribution of indicators in liver tissues

**Figure 1:**
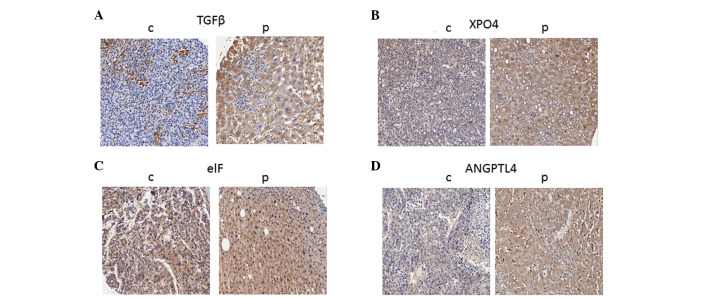
Immunostaining images for TGFβ1, XPO4, ANGPTL4 and elF5A2 in carcinoma and paracarcinoma tissues. c indicates the carcinoma tissue and p indicates the paracarcinoma tissue.

The four indicators, XPO4, TGFβ1, ANGPTL4 and elF5A2, were found to be located within the cellular cytoplasm by the immunostaining method (Fig. 1). The data shown in Table I are based on the immunohistochemical staining levels. The values in the table were obtained from the percentage of positive staining in the same samples among TMAs. With regard to tissue distribution of the 4 indicators, they were all expressed in cancerous and paracancerous liver tissues. They were all found at a significantly higher density in the paracancerous tissues than in the cancerous liver tissues (Table I).

### Correlation in expression among these indicators

The correlation in expression of XPO4 between the cancerous and paracancerous liver tissue was positive (CC=0.304, P<0.001). Expression of XPO4 in the cancerous liver tissue was positively correlated with expression of TGFβ1 (CC=0.126, P=0.047) in paracancerous liver tissue, expression of ANGPTL4 (CC=0.506, P=0.000) in cancerous liver tissue, expression of ANGPTL4 (CC=0.199, P=0.002) in para-cancerous liver tissue and expression of elF5A2 (CC=0.194, P=0.002) in paracancerous liver tissue (Table II). The correlation in expression of ANGPTL4 between the cancerous and paracancerous liver tissue was positive (CC=0.282, P<0.001). Expression of ANGPTL4 in the cancerous liver tissue was positively correlated with expression of XPO4 (CC=0.506, P<0.001) in carcinoma liver tissue, expression of elF5A2 (CC=0.469, P<0.001) in carcinoma liver tissue and expression of elF5A2 (CC=0.245, P<0.001) in paracancerous liver tissue. The correlation in expression of elF5A2 between cancerous and paracancerous liver tissues was positive (CC=0.371, P<0.001). Expression of elF5A2 in the cancerous liver tissue was positively correlated with expression of XPO4 (CC=0.478, P<0.001) in carcinoma liver tissue. These results suggest that the expression of these four indicators is internally connected and there is modulation between each of them.

### Correlation between expression of indicators and pathological information

#### Indicators and tumor size

In patients with multi-nodular tumors, the tumor samples were obtained from the largest tumor. The statistical results revealed that the expression of TGFβ1 in paracancerous liver tissue was significantly positively correlated with tumor size (CC=0.147, P=0.021, n=248; Table III). The other 7 parameters, e.g., TGFβ1 in cancerous liver tissue, and XPO4 in cancerous liver tissue, had no significant correlation with tumor size.

*Indicators and blood vessel invasion* The statistical results revealed that all indicators in cancerous and paracancerous liver tissues had no significant correlation with blood vessel invasion (Table IV).

#### Indicators and pathological classification (differentiation)

The patients were divided into two categories according to the Edmondson classification; high differentiation (I, II, I–II) and low differentiation (II–III, III, IV). The statistical results revealed that all indicators exhibited higher expression levels in the low differentiation group than in the high differentiation group (Table V). XPO4 in cancerous liver tissue (CC=0.143, P=0.035) and TGFβ1 (CC=0.195, P=0.004) in paracancerous liver tissue were significantly correlated with tumor differentiation.

*Indicators and tumor T classification* The statistical results revealed that expression of TGFβ1 in both cancerous and paracancerous tissues (CC=0.402, P=0.000; CC=0.299, P=0.000, respectively) was positively correlated with T classification; expression of ANGPTL4 in cancerous and paracancerous liver tissues (CC=0.125, P=0.049; CC=0.142, P=0.025, respectively) was positively correlated with T classification and that the expression of elF5A2 in paracancerous liver tissues (CC=0.127, P=0.047) was positively correlated with T classification.

#### Indicators and survival function

**Figure 2:**
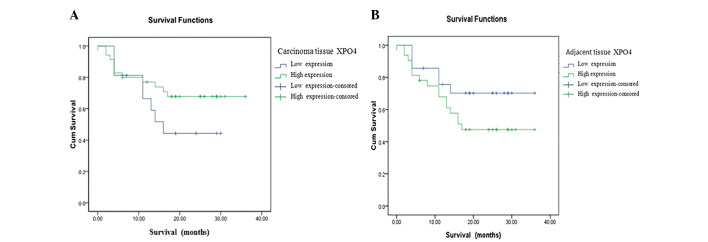
Kaplan-Meier analysis of XPO4 correlated with patient survival function. (A) Kaplan-Meier analysis indicated that expression of XPO4 in carcinoma tissue did not correlate with overexpression or underexpression (P=0.202), and that survival rates of XPO4-overexpressing patients were higher than those of patients with XPO4 underexpression. (B) Kaplan-Meier analysis indicated that expression of XPO4 in adjacent tissue did not correlate with overexpression or underexpression (P=0.139), and that survival rates of XPO4-overexpressing patients were lower than those of patients with XPO4 underexpression.

**Figure 3:**
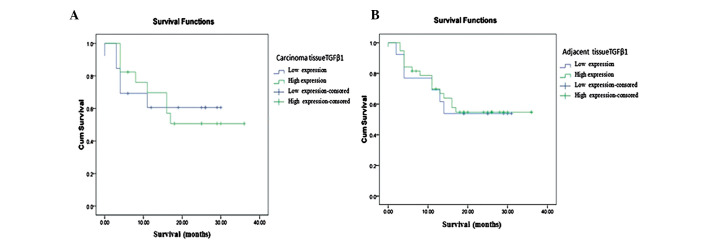
Kaplan-Meier analysis of TGFβ1 in adjacent tissue correlated with patient survival. Expression of TGFβ1 in carcinoma tissue (A) was not correlated with overexpression and underexpression (P=0.954). The results indicated that expression of TGFβ1 in adjacent tissue (B) was not correlated with overexpression and underexpression (P=0.884).

Kaplan-Meier analysis indicated that the expression of XPO4 in carcinoma tissue did not correlate with survival function in overexpression and underexpression (P=0.202). The survival plot indicated that survival rates in patients with XPO4 overexpression were higher than those in patients with XPO4 underexpression (Fig. 2A). Expression of XPO4 in adjacent tissue did not correlate with overexpression or underexpression (P=0.139). The survival plot indicated that survival rates in patients with XPO4 overexpression in adjacent tissues were lower than those in patients with XPO4 underexpression. These results suggested that higher expression of XPO4 in cancerous liver tissue was indicative that the patient would have a better prognosis and increased survival rate. However, higher concentrations of XPO4 in paracancerous liver tissue suggested a worse prognosis (Fig. 2B). Furthermore, expression of TGFβ1 in carcinoma tissue did not correlate with overexpression or underexpression (P=0.954). The survival figure indicated that patients who were positive for TGFβ1 in cancerous liver tissue had a better prognosis than those who were negative for TGFβ1 in cancerous liver tissue (Fig. 3). Other factors, e.g., ANGPTL4 and ELF, were not correlated with overexpression or underexpression in either of the cancerous and adjacent tissues.

## Discussion

In the present study, we employed multiple techniques, including the use of qPCR, immunostaining and TMAs, as well as histology and pathology analysis, to undertake a study to evaluate XPO4, TGFβ1, ANGPTL4 and elF5A2 in carcinoma and paracarcinoma tissues from 280 liver cancer patients. Our results revealed that all four indicators were located in the cytoplasm and distributed in both cancerous and paracancerous liver tissues. Generally, there were higher levels of these indicators in paracancerous than in cancerous liver tissue. The correlation analysis results further revealed that expression levels of these four indicators were correlated and modulated amongst each other. In connection with patients’ clinical and revisit information, statistical results revealed that TGFβ1 levels in paracancerous liver tissue was positively correlated with the tumor size. Higher levels of TGFβ1 in paracancerous liver tissue were always associated with bigger liver tumors. XPO4 in cancerous liver tissue and TGFβ1 in paracancerous liver tissue were positively correlated with tumor differentiation. TGFβ1, ANGPTL4 and elF5A2 were also positively correlated with the T classification of tumors. Additionally, a higher expression of XPO4 in cancerous liver tissue suggested that the patient would have a better prognosis and survival rate. However, higher XPO4 levels in paracancerous liver tissue suggested a worse prognosis. These results suggest that XPO4 may be a potential biomarker to assess HCC prognosis and differentiation.

In the present study, we performed experimental and statistical work in cancerous and paracancerous materials from 280 patients suffering from liver cancer. All patient materials were collected and preserved according to standard protocols (see Patients and methods). In order to reduce the experimental error/variance obtained by processing different tissue samples using immunostaining, we selected the method of TMAs to equalize the experimental condition among different samples. TMA is an innovative type of specimen slide with multiple samples on a single slide, which was first described in 1986 (17,18). In our study, each slide integrated 200 tissue specimens on a single slide as 500 dots, which represented multiple tissues, pathological types, patients and stages, as well as control samples, aiding in the creation of a low margin for error. This method allows for high throughput of data of multiple patient cases and control samples simultaneously. Thus reagent concentrations were identical for each case, as were incubation times, temperatures and wash conditions. This greatly improves the accuracy of the results, leading to more confident conclusions (19). Each experiment was also repeated in three slides of TMAs. Data averages were used for analysis.

We have been aware of the result complicity from different patients and made numerous attempts to summarize our results in a realistic way. It is conventional to take the middle point as a standard to perform analysis in survival function. However, there is no meaning because the positive probability of indicators displays skew distribution. The 25th percentile was therefore eventually taken for correlation analysis in our study. The suggestion given by this analysis is rational and it is close to clinic observation by current knowledge.

TGFβ is a multipotent polypeptide which regulates cell proliferation, differentiation and apoptosis and deters tumor growth (20). Within the tumor micro-environment, TGFβ is produced by liver cancer cells. It is located within the cytoplasm, as shown in our results and consistently in a number of previous studies (3,5). However, as a natural response to the hypoxic and inflammatory conditions that occur during tumor progression, high production levels of TGFβ1 in our study were unexpectedly found in paracancerous, but not cancerous, liver tissue. Moreover, TGFβ1 in cancerous or paracancerous liver tissue had no connection with either tumor vessel invasion or lymph metastasis. These results were inconsistent with previous results which demonstrated that the overexpression of TGFβ1 is associated with a high incidence of distant metastasis and that TGFβ1 promotes vascular invasion by activating β1 integrin (10,21). Additionally, patients positive for TGFβ1 in the cancerous tissue were suggested to have a better prognosis, as is consistent with a previous report (6). This result may partly support the current concept for a dual role of the TGFβ signaling pathway in hepatocellular cancer suppression and progression of differentiation (3,6,22,23).

TGFβ receptors phosphorylate Smad3 and induce its nuclear import, then regulate gene transcription. Smad3 returns to the cytoplasm to propagate further cycles of signal transduction. In HCCs, Smad3 and its phosphorylation relatives have been suggested to be the predictors of prognosis in patients with liver cancer and also serve as the biomarkers to identify patients with a high risk of recurrence (5). Smad3 is exported via XPO4. XPO4 is therefore in control of Smad3 signaling as well as protein synthesis (7,8). Consistent with a previous study (9), our results also demonstrated that XPO4 was present in cancerous and paracancerous liver tissues, with a higher density of XPO4 in paracancerous tissue. Our results found that TGFβ1, but not XPO4 (9), in paracancerous tissue was significantly positively correlated with tumor size and histopathological classification. Moreover, our results also suggested that high XPO4 in cancerous tissue resulted in a good prognosis. This is consistent with a previous study which indicated that downregulation of XPO4 resulted in a poor prognosis (9). Our results in an adequate sample size emphasized again that XPO4 may be involved in the progression of human HCC and may serve as a potential biomarker to evaluate the condition. A further validation study is suggested on a bigger sample size. Additionally, assessment of tumor behavior in HCC cell lines with or without rescuing XPO4 may confirm the therapeutic role of XPO4 in HCC.

elF5A2 is amplified in human tumors, is required for proliferation of XPO4-deficient tumor cells and promotes HCC in mice (7). Our results provided evidence of correlation among these four indicators in HCC. Production of elF5A2 in paracancerous tissue is significantly positively associated with cancer histopathological classification. Previous studies have also revealed that the induction of angiopoietin-like 4 (ANGPTL4) by TGFβ, via the Smad signaling pathway, is critical for the trans-endothelial passage of tumor cells resulting in tumor metastasis (14). Further studies have revealed that ANGPTL4 regulates endothelial cell junction organization (24) and pericyte coverage (11), resulting in disruption in endothelial cell-cell junctions (25). We therefore carried out the rational measurement of ANGPTL4 in the cancerous and paracancerous liver tissues in a variety of patients. Our results showed that there was no significant correlation between ANGPTL4 and vessel or lymphatic invasion. It is inconsistent with previous reports that expression of ANGPTL4 was statistically correlated with the degree of differentiation, lymphatic invasion and venous invasion (12,26). Our results provided evidence that ANGPTL4 is not a metastasis-inducing factor in HCC.

In summary, the results of the present study revealed that XPO4, TGFβ1, ANGPTL4 and elF5A2 were present in both cancerous and paracancerous liver tissues, and that they were closely correlated with each other. TGFβ1 in paracancerous liver tissue was positively correlated with the tumor size. XPO4 in cancerous liver tissue and TGFβ1 in paracancerous liver tissue were positively associated with tumor differentiation. Meanwhile, TGFβ1, ANGPTL4 and elF5A2 were significantly correlated with the T classification of tumors. Of these four indicators, XPO4 appears to be a potential biomarker to evaluate HCC.

## Figures and Tables

**Table I:** Expression of XPO4, TGFβ1, ANGPTL4 and elF5A2.

Indicator	Carcinoma tissue	Adjacent tissue	P-value^a^
XPO4	0.800±0.194	0.855±0.113	0.000
TGFβ1	0.256±0.284	0.502±0.312	0.000
ANGPTL4	0.723±0.247	0.817±0.173	0.000
elF5A2	0.770±0.176	0.814±0.141	0.000

a Paired-sample t-test. P<0.05 was considered to indicate a statistically significant difference. XPO4, TGFβ1, ANGPTL4 and elF5A2 expression in carcinoma tissues was significantly lower than that in adjacent tissues.

**Table II:** Correlation of XPO4, TGFβ1, ANGPTL4 and elF5A2 in carcinoma tissues and adjacent tissues.

Tissue and indicator, statistical test	Carcinoma tissue XPO4	Adjacent tissue XPO4	Carcinoma tissue TGFβ1	Adjacent tissue TGFβ1	Carcinoma tissue ANGPTL4	Adjacent tissue ANGPTL4	Carcinoma tissue elF5A2	Adjacent tissue elF5A2	TNM
Carcinoma tissue XPO4									
Correlation	1	0.304^b^	0.038	0.126^a^	0.506^b^	0.199^b^	0.478^b^	0.194^b^	0.052
Sig. (2-tailed)	-	0.000	0.562	0.047	0.000	0.002	0.000	0.002	0.415
n	261	250	239	251	258	249	257	245	249
Adjacent tissue XPO4									
Correlation	0.304^b^	1	0.029	0.142^a^	0.118	0.224^b^	0.106	0.215^b^	0.042
Sig. (2-tailed)	0.000	-	0.663	0.023	0.062	0.000	0.096	0.001	0.512
n	250	257	234	256	249	254	248	252	247
Carcinoma tissue TGFβ1									
Correlation	0.038	0.029	1	0.467^b^	0.104	0.172^b^	0.047	0.065	0.402^b^
Sig. (2-tailed)	0.562	0.663	-	0.000	0.109	0.008	0.468	0.329	0.000
n	239	234	241	234	238	233	237	230	230
Adjacent tissue TGFβ1									
Correlation	0.126^a^	0.142^a^	0.467^b^	1	0.085	0.228^b^	0.066	0.103	0.299^b^
Sig. (2-tailed)	0.047	0.023	0.000	-	0.181	0.000	0.299	0.104	0.000
n	251	256	234	258	250	254	249	251	247
Carcinoma tissue ANGPTL4									
Correlation	0.506^b^	0.118	0.104	0.085	1	0.282^b^	0.469^b^	0.245^b^	0.125^a^
Sig. (2-tailed)	0.000	0.062	0.109	0.181	-	0.000	0.000	0.000	0.049
n	258	249	238	250	260	248	259	244	248
Adjacent tissue ANGPTL4									
Correlation	0.199^b^	0.224^b^	0.172^b^	0.228^b^	0.282^b^	1	0.239^b^	0.477^b^	0.142^a^
Sig. (2-tailed)	0.002	0.000	0.008	0.000	0.000	-	0.000	0.000	0.025
n	249	254	233	254	248	256	247	252	247
Carcinoma tissue elF5A2									
Correlation	0.478^b^	0.106	0.047	0.066	0.469^b^	0.239^b^	1	0.371^b^	0.050
Sig. (2-tailed)	0.000	0.096	0.468	0.299	0.000	0.000	-	0.000	0.437
n	257	248	237	249	259	247	259	244	248
Adjacent tissue elF5A2									
Correlation	0.194^b^	0.215^b^	0.065	0.103	0.245^b^	0.477^b^	0.371^b^	1	0.127^a^
Sig. (2-tailed)	0.002	0.001	0.329	0.104	0.000	0.000	0.000		0.047
n	245	252	230	251	244	252	244	252	244
TNM									
Correlation	0.052	0.042	0.402^b^	0.299^b^	0.125^a^	0.142^a^	0.050	0.127^a^	1
Sig. (2-tailed)	0.415	0.512	0.000	0.000	0.049	0.025	0.437	0.047	-
n	249	247	230	247	248	247	248	244	256

a Correlation is considered to be statistically significant at the 0.05 level (2-tailed).

b Correlation is considered to be statistically significant at the 0.01 level (2-tailed). The correlation in expression of XPO4 between the cancerous and paracancerous liver tissue was positive (CC=0.304, P<0.001). Expression of XPO4 in the cancerous liver tissue was positively correlated with expression of TGFβ1 (CC=0.199, P=0.047) in paracancerous liver tissue, expression of ANGPTL4 (CC=0.506, P=0.000) in cancerous liver tissue, expression of ANGPTL4 (CC=0.199, P=0.002) in paracancerous liver tissue and expression of elF5A2 (CC=0.194, P=0.002) in paracancerous liver tissue, respectively. These results suggest that expression of these four indicators is internally connected and that there is modulation between each of them. TNM, TNM classification of malignant tumors.

**Table III:** Correlation between indicators and tumor size.

Statistical test	Carcinoma tissue XPO4	Adjacent tissue XPO4	Carcinoma tissue TGFβ1	Adjacent tissue TGFβ1	Carcinoma tissue ANGPTL4	Adjacent tissue ANGPTL4	Carcinoma tissue elF5A2	Adjacent tissue elF5A2
Tumor size								
Correlation	−0.122	0.066	0.051	0.147^a^	−0.116	0.089	−0.040	0.053
Sig. (2-tailed)	0.054	0.300	0.438	0.021	0.068	0.164	0.529	0.407
n	250	247	232	248	249	247	248	244

a Correlation is considered to be statistically significant at the 0.05 level (2-tailed).

b Correlation is considered to be statistically significant at the 0.01 level (2-tailed).

**Table IV:** Correlation between XPO4, TGFβ1, ANGPTL4 and elF5A2 expression and vascular invasion in carcinoma tissue and adjacent tissue.

Indicator	Tissue type	Vascular invasion (yes)	Vascular invasion (no)	P-value
XPO4	Carcinoma tissue	0.689±0.317	0.803±0.185	0.313
	Adjacent tissue	0.806±0.174	0.855±0.111	0.417
TGFβ1	Carcinoma tissue	0.259±0.300	0.257±0.281	0.984
	Adjacent tissue	0.539±0.227	0.502±0.311	0.724
ANGPTL4	Carcinoma tissue	0.780±0.132	0.723±0.247	0.465
	Adjacent tissue	0.889±0.042	0.816±0.172	0.210
elF5A2	Carcinoma tissue	0.760±0.145	0.769±0.174	0.861
	Adjacent tissue	0.867±0.070	0.812±0.143	0.258

The statistical results revealed that all indicators in cancerous and paracancerous liver tissue had no significant correlation with blood vessel invasion.

**Table V:** Association of XPO4, TGFβ1, ANGPTL4 and elF5A2 expression with differentiation.

Indicator	Tissue type	High differentiation	Low differentiation	P-value
XPO4	Carcinoma tissue	0.793±0.195	0.850±0.150	0.035
	Adjacent tissue	0.849±0.115	0.881±0.106	0.054
TGFβ1	Carcinoma tissue	0.312±0.290	0.303±0.269	0.867
	Adjacent tissue	0.540±0.285	0.658±0.245	0.003
ANGPTL4	Carcinoma tissue	0.751±0.213	0.737±0.211	0.643
	Adjacent tissue	0.833±0.148	0.842±0.121	0.689
elF5A2	Carcinoma tissue	0.775±0.170	0.800±0.106	0.253
	Adjacent tissue	0.816±0.152	0.845±0.082	0.080

Correlation regression analysis indicated that expression of TGFβ1 in adjacent tissue and XPO4 in carcinoma tissue were significantly correlated with differentiation. XPO4 of carcinoma tissue, CC=0.143, P=0.035; TGFβ1 of adjacent tissue, CC=0.195, P=0.004. CC, correlation coefficient.
